# 

**Published:** 1922-06

**Authors:** 


### THE BRISTOL
### Medico-Chirurgical Journal.

A JOURNAL OF THE MEDICAL SCIENCES FOR THE WEST OF ENGLAND AND SOUTH WALES.

*PUBLISHED UNDER THE AUSPICES OF*

*THE BRISTOL MEDICO-CHIRURGICAL SOCIETY.*

EDITOR:
P. WATSON-WILLIAMS, M.D.

WITH WHOM ARE ASSOCIATED

JAMES SWAIN, C.B., C.B.E., M.S., M.D., J. LACY FIRTH, M.S., M.D.,
CAREY COOMBS, M.D.

J. A. NIXON, C.M.G., M.D., Assistant Editor.

EDITORIAL SECRETARY:
A. L. FLEMMING, M.B., Ch.B.

"Scire est nescire, nisi id me
Scire alius sciret."

VOL. XXXIX.

53
2031
D

Library of Congress

541396

FEB 1 1923

SMITHSONIAN DEPOSIT

BRISTOL: J. W. ARROWSMITH LTD.
LONDON: SIMPKIN, MARSHALL, HAMILTON, KENT & Co. LIMITED.
1922.

R31
.B8

### INDEX.

Ackland, W. R.—Oral sepsis as a source of systemic infections, 76.

Adenoids, The routine of operations for, 52.

Appendicitis, Diagnosis of malignant disease of cæcum from chronic and subacute—C. A. Morton, 82.

**Books, Reviews of**—

Abdomen, The acute—Dr. Z. Cope, 91.
Abrahams, A.—Urinary diagnosis, 92.
Bowel diseases in the tropics—Sir L. Rogers, 51.
Bradby, M. K.—The logic of the unconscious mind, 102.
Circulatory disease, The early symptoms and treatment of—Dr. R. M. Wilson, 48.
Cope, Dr. Z.—The acute abdomen, 91.
Crile, Dr. G. W.—A physical interpretation of shock, 49.
Davis, Dr. H.—Skin diseases, 96.
Dental mechanics—H. Osborn, 102.
Dermatology, Introduction to—Dr. N. Walker, 95.
Electro-therapeutics—Dr. F. H. Humphris, 96.
Electrotherapy—Dr. W. J. Turrell, 100.
Fevers, Manual of—Dr. C. B. Ker, 101.
Fractures—E. W. H. Groves, 48.
Gout, Physiology of—Dr. P. Wilde, 96.
Groves, E. W. H.—Fractures, 48.
Humphris, Dr. F. H.—Electro-therapeutics 96.
Immunization, Therapeutic—Dr. W. F. Robertson, 94.
Journal of metabolic research, 50.
Ker, Dr. C. B.—Manual of fevers, 101.
Larynx, Intrinsic cancer of—Dr. I. Moore, 50.
Luke, Dr. T. D.—Physio-therapeutics, 100.
Monrad-Krohn, Dr. G. H.—Clinical examination of the nervous system, 48.
Moore, Dr. I.—Intrinsic cancer of larynx, 50.
Moynihan, Sir B.—The spleen and some of its diseases, 91.
Nervous system, The clinical examination of the—Dr. G. H. Monrad-Krohn, 48.
Operative surgery, Aids to—H. C. Orrin, 93.
Orbit, The anatomy of the human—Dr. S. E. Whitnall, 97.
Orrin, H. C.—Aids to operative surgery, 93.
Osborn, H.—Golden rules of dental mechanics, 102.
Peripheral nerve injuries of warfare, The surgery of the—H. Platt, 92.
Peripheral spinal nerves, Injuries to the—Sir H. J. Stiles, 98.
Physio-therapeutics—Dr. T. D. Luke, 100.

**Books, Reviews of** (*continued*)—

Platt, H.—The surgery of peripheral nerve injuries of warfare, 92.
Psychopathic diseases—Dr. B. Sidis, 93.
Quervain, F. de—Clinical surgical diagnosis, 47.
Riviere, Dr. C.—The early diagnosis of tubercle, 47.
Robertson, Dr. W. F.—Therapeutic immunization, 94.
Rogers, Sir L.—Bowel diseases in the tropics, 51.
Sherren, J.—Surgery of stomach and duodenum, 92.
Shock, A physical interpretation of—Dr. G. W. Crile, 49.
Sidis, Dr. B.—Psychopathic diseases, 93.
Skin diseases—Dr. H. Davis, 96.
Sorapure, Dr. V. E.—Index of therapeutics, 99.
Spleen and some of its diseases—Sir B. Moynihan, 91.
Stiles, Sir H. J.—Injuries of the peripheral spinal nerves, 98.
Stomach, Surgery of the—J. Sherren, 92.
Surgical diagnosis, Clinical—F. de Quervain, 47.
Therapeutics, Index of—Dr. V. E. Sorapure, 99.
Tubercle, The early diagnosis of—Dr. C. Riviere, 47.
Turrell, Dr. W. J.—Electrotherapy, 100.
Unconscious mind, The logic of the—M. K. Bradby, 102.
Urinary diseases, A guide to—Dr. A. Abrahams, 92.
Walker, Dr. N.—Introduction to dermatology, 95.
Wilde, Dr. P.—The physiology of gout, 96.
Wilson, Dr. R. M.—Early symptoms and treatment of circulatory diseases, 48.
Whitnall, Dr. S. E.—The anatomy of the human orbit, 97.

Cæcum, Malignant disease of, Diagnosis between chronic and subacute appendicitis—C. A. Morton, 82.

Carleton, Dr. H. H.—A physiological conception of prolonged hysterical conditions, 36.

Clarke, Dr. C.—Renal efficiency, 13.

Davies, Dr. D. S., the Medical Officer of Health, 69.

Diabetes, Insulin in the treatment of, 103.

Editorial notes, 52, 103.

2/XII/24

Hadfield, Dr. G.—Renal efficiency, 28.
Hysterical conditions, Physiological conception of prolonged—Dr. H. H. Carleton, 36.

Insulin in treatment of diabetes, 103.

Library of the Bristol Medico-Chirurgical Society, 110.
Local medical notes, 67, 115.
Loveday, Mr. T., appointment of, 103.

Medical officer of health, Bristol's, 69.
Medical school incorporation, 108.
Medicine and psychology—Sir J. H. Parsons, 1.
Morton, C. A.—The differential diagnosis of malignant disease of the cæcum from chronic and subacute appendicitis, 82.

Obituary, 57, 112.
Oral sepsis as a source of systemic infections—W. R. Ackland, 76.

Parsons, Sir J. H.—Medicine and psychology, 1.
Postgraduate instruction, 106.
Psychology, Medicine and—Sir J. H. Parsons, 1.

Renal efficiency, Discussion on, 13.

Smith, Dr. R. S., Obituary notice of, 57.
Stack, Dr. E. H. E., Obituary notice of, 112.
Symes, Dr. J. O.—Renal efficiency, 25.

Tonsils, The routine of operations for, 52.

Wills, Mr. H. H., death of, 54.

J. W. Arrowsmith Ltd

Publishers Bristol

### CONTENTS OF VOLUME XXXIX.

*Page*

Medicine and Psychology. By Sir John Herbert Parsons, C.B.E., F.R.C.S., F.R.S. .. .. .. .. .. .. .. 1

Discussion on Renal Efficiency. By Cecil Clarke, M.D. Lond., M.R.C.P., J. O. Symes, M.D. Lond., G. Hadfield, M.D. Lond. .. 13

A Physiological Conception of Prolonged Hysterical Conditions. By H. H. Carleton, M.A., M.D. Oxon. .. .. .. .. 36

The Bristol Medical Officer of Health .. .. .. .. .. 69

Oral Sepsis as a Source of Systemic Infections. By W. R. Ackland, M.R.C.S., L.D.S., M.D.S. .. .. .. .. .. .. 76

The Differential Diagnosis of Malignant Disease of the Cæcum from Chronic and Subacute Appendicitis. By Charles A. Morton, F.R.C.S. .. .. .. .. .. .. .. .. 82

Reviews of Books .. .. .. .. .. .. 47, 91

Editorial Notes .. .. .. .. .. .. .. 52, 103

The Library of the Bristol Medico-Chirurgical Society .. 110

Obituary .. .. .. .. .. .. .. .. 57, 112

Local Medical Notes .. .. .. .. .. .. 67, 115

### Bristol Medico-Chirurgical Society.

### PAST PRESIDENTS.

1874—75. FREDERICK BRITTAN, M.D., B.A. Dub.
1875—76. ROBERT WILLIAM COE.
1876—77. SAMUEL MARTYN, M.D. Ed.
1877—78. AUGUSTIN PRICHARD, M.D. Berlin.
1878—79. HENRY EDWARD FRIPP, M.D. Würzburg.
1879—80. WILLIAM MICHELL CLARKE.
1880—81. JOSEPH GRIFFITHS SWAYNE, M.D. Lond.
1881—82. EDWARD LONG FOX, M.D. Oxon.
1882—83. JAMES GEORGE DAVEY, M.D. St. And.
1883—84. WILLIAM JOHNSTONE FYFFE, M.D., B.A. Dub.
1884—85. GEORGE FORSTER BURDER, M.D. Aberd.
1885—86. WILLIAM HENRY SPENCER, M.D., M.A. Cantab.
1886—87. FRANCIS POOLE LANSDOWN.
1887—88. LEMUEL MATTHEWS GRIFFITHS.
1888—89. ROBERT SHINGLETON SMITH, M.D., B.Sc. Lond.
1889—90. NELSON CONGREVE DOBSON, Ch.M. Bristol
1890—91. SAMUEL HENRY SWAYNE.
1891—92. FRANCIS RICHARDSON CROSS, M.B.Lond., LL D.Bristol.
1892—93. EDWARD MARKHAM SKERRITT, M.D., B.S., B.A. Lond.
1893—94. JAMES GREIG SMITH, M.B., C.M., M.A. Aberd., F.R.S.E.
1894—95. ALFRED JAMES HARRISON, M.B. Lond.
1895—96. ARTHUR WILLIAM PRICHARD.
1896—97. ALFRED EDWARD AUST LAWRENCE, M.D., C.M. Aber .
1897—98. JOHN EDWARD SHAW, M.B., C.M. Ed.
1898—99. ROBERT ROXBURGH, M.B. Ed.
1899—00. WILLIAM HENRY HARSANT.
1900—01. DAVID SAMUEL DAVIES, M.D., Lond.
1901—02. BARCLAY JOSIAH BARON, M.B., C.M. Ed.
1902—03. GEORGE MUNRO SMITH, M.D. Bristol.
1903—04. JAMES PAUL BUSH, C.M.G., C.B.E., Ch.M. Bristol.
1904—05. REGINALD EAGER, M.D. Lond.
1905—06. JOHN DACRE.
1906—07. JAMES TAYLOR.
1907—08. HENRY WALDO, M.D., C.M. Aberd.
1908—09. JOHN MICHELL CLARKE, M.D., M.A. Cantab.
1909—10. JAMES SWAIN, C.B., C.B.E., M.D., M.S. Lond.
1910—11. BERTRAM MITFORD HERON ROGERS, M.D., B.Ch., B.A. Oxon.
1911—12. CHARLES ALEXANDER MORTON.
1912—13. WALTER CARLESS SWAYNE, M.D., B.S. Lond. and Bristol.
1913—14. PATRICK WATSON-WILLIAMS, M.D. Lond.
1914—15. WILLIAM HARRY CHRISTOPHER NEWNHAM, M.A., M.B. Cantab.
1915—16 GEORGE PARKER, M.A., M.D. Cantab.
1916—17 FRANCIS HENRY EDGEWORTH, M.A., M.D. Cantab., D.Sc. Lond.
1917—18 FREDERICK LACE.
1918—19 ROBERT GUTHRIE POOLE LANSDOWN, M.D., B.S. Durh.
1919—20 I. WALKER HALL, M.D., Ch.B. Vict.
1920—21 L. E. V. EVERY-CLAYTON, M.D. Lond.

1922.

### LIST OF SUBSCRIBERS

TO THE

### Bristol Medico-Chirurgical Journal.

### HONORARY MEMBERS OF THE SOCIETY.

| | |
|---|---|
| Griffiths, L. M. ... ... ... ... | 11 Pembroke Road, Clifton. |
| Owen, Sir Isambard, D.C.L., M.D. | Abbey Gate, Beach Road, Weston-super-Mare. |

### SUBSCRIBERS.

*Those marked * are Members of the Society.*

| | |
|---|---|
| Ackland, J. McKno ... ... ... ... ... ... | 1 Barnfield Crescent, Exeter. |
| *Ackland, W. R., M.D.S. Brist. ... ... ... | 5 Rodney Place, Clifton. |
| *Adams, A. W., M.B., B.Sc. Lond. ... ... ... | 9 Mortimer Road, Clifton. |
| Adye, W. J. A. ... ... ... .. ... ... ... | St. Margarets, Bradford-on Avon, Wilts. |
| *Alexander, D. A., M.B., Ch.B. Vict. ... ... | 30 Berkeley Square, Clifton. |
| *Askins, R. A., M.D., B.Ch., B.A.O. Dubl.... | Guildhall, Bristol. |
| *Aubrey, T., M.B. Lond. ... ... ... ... ... | Bitton, near Bristol. |
| *Baker, Lily A., M.D., B.Ch., B.A.O., R.U.I.... | 2 Harley Place, Clifton. |
| *Ball, E. J., M.A., Ph.D., Heidl. ... ... ... | 120 Pembroke Road, Bristol. |

### LIST OF SUBSCRIBERS.

| | |
|---|---|
| *BARBER, M. C., M.B., Ch.B. Brist. ... ... ... | Ingledene, High Street, Staple Hill, Bristol. |
| *BARKER, G. H., M.D., B.Sc. Lond. ... ... ... | 124 Redland Road, Bristol. |
| *BARTHOLOMEW, E. U. ... ... ... ... ... ... | 7 Clifton Hill, Clifton. |
| *BERGIN, F. G. ... ... ... ... ... ... ... ... | 31 Oakfield Road, Clifton. |
| BERNARD, C. ... ... ... ... ... ... .. .. | 704 Fishponds Road, Fishponds, Bristol. |
| BERRY, F. C., M.D., B.Ch., B.A. Dub. ... ... | Locarno, Burnham, Somerset. |
| *BLACKER, A. E.... ... ... ... ... ... ... ... | 20 Victoria Square, Clifton. |
| BLACKETT, J. F., M.D., B.S. Lond. ... ... ... | Hamsterley, Bloomfield Park, Bath. |
| *BLAGG, ARTHUR F., M.D. Durh.... ... ... ... | 28 Caledonia Place, Clifton. |
| *BODMAN, C. O., M.D. Durh. ... ... ... ... | 17 Upper Belgrave Road, Clifton. |
| *BODMAN, J. HERVEY, M.D. Lond. ... ... ... | 9 Whiteladies Road, Clifton. |
| *BOWKER, G. E., M.D. Edin.... ... ... ... ... | 11 North Parade, Bath. |
| *BRADLEY, E. J., M.B. ... ... ... ... ... ... | Bristol General Hospital. |
| BRASHER, C. W. J., M.D. Brist. ... .. ... | 23 Victoria Square, Clifton. |
| *BRAY, G., Lt.-Col., *D.S.O.* ... ... ... ... | 10 Miles Road, Clifton. |
| *BRISTOWE, H. C., M.D. Lond. ... ... ... ... | Wrington, Somerset. |
| *BROWN, WILLIAM, M.D., C.M. Glasg. ... ... | Park View, Fishponds, Bristol. |
| *BUCKMASTER, G. A., M.A., M.D. Oxon. ... ... | University of Bristol. |
| *BURDON-COOPER, J., M.D., B.Sc. Durh. ... ... | 12 The Circus, Bath. |
| *BUSH, J. PAUL, *C.M.G.*, *C.B.E.*, Ch.M. Brist. | Vyvyan House, Clifton. |

| | |
|---|---|
| *CAIRNS, F. J. ... ... ... ... ... ... ... ... | Devon House, Whitehall Road, Bristol. |
| *CALDWELL, J. R., M.B., Ch.B. St. Andrews ... | Sunnyside, Montrose, Scotland. |
| *CAREY, R. S., *O.B.E.*, B.A. Cantab. ... ... | 502 Bath Road, Brislington, Bristol. |
| *CARLETON, H. H., M.A., M.D. B.Ch. Oxon. | 72 Pembroke Road, Clifton. |
| *CARLING, A., M.A., M.B., B.C. Cantab. ... ... | 12 Great George Street, Bristol. |
| *CARWARDINE, THOMAS, M.B., M.S. Lond. ... | 16 Victoria Square, Clifton. |
| *CAVE, EDWARD J., M.D. Lond. ... ... ... ... | 20 The Circus, Bath. |
| *CHAMBERS, E. R. ... ... ... ... ... ... | 9 Clifton Park, Clifton. |
| *CHARLES, J. R., M.A., M.D., B.C. Cantab. ... | 5 Rodney Place, Clifton. |
| *CHITTY, H., M.S. Lond. ... ... ... ... ... | 46 Pembroke Road, Clifton. |
| *CHRISTOFFERSON, P. E., M.B., Ch.B. Brist. ... | 8 Lansdown Place, Clifton. |
| CHUTE, HENRY M. ... ... ... ... ... ... ... | King William's Town, S. Africa. |
| *CLARKE, CECIL, M.B., B.S. Lond. ... ... ... | 3 Fosseway, Clifton. |
| *CLARKE, R. C., *O.B.E.*, M.B., Ch.B. Brist. ... | 29 Victoria Square, Clifton. |
| *COATES, VINCENT M., *M.C.*, M.A., M.D B.C. Cantab. .. ... ... ... ... ... ... | 10 The Circus, Bath. |
| CONNELLAN, P. S. ... ... ... ... ... ... ... | 12 Bloomsbury Street, London, W.C.1. |
| *COOMBS, CAREY, M.D. Lond. ... ... ... ... | 3 Pembroke Road, Clifton. |
| *COOPER, WILLIAM RUSSELL ... ... ... ... | 13 Westfield Park, Redland, Bristol. |
| *CORFIELD, C., M.D. Durh. ... ... ... ... | 5 Kensington Place, Clifton. |
| COSSHAM, W. R., M.D., C.M. Aberd.... ... ... | Wellesley House, Cirencester. |
| COULTER, R. J., M.B. Dub. ... ... ... ... ... | Ferncliffe, Stow Park Crescent, Newport, Mon. |
| CRIDLAND, A. B. ... ... ... ... ... ... ... | Salisbury House, Chapel Ash, Wolverhampton. |
| *CROSS, F. R., M.B. Lond., LL.D. Brist. ... | Worcester House, Clifton. |
| *CROSSMAN, F. W., M.B. Durh. ... ... ... ... | White's Hill, Hambrook, near Bristol. |
| *CROUCH, C. PERCIVAL, M.B. Lond. ... ... ... | "Penquarry," Weston-super-Mare. |
| *CUMMINS, Col. S. LYLE, *C.B.*, *C.M.G.*, M.D., N.U.I. ... ... ... ... ... ... ... | Welsh National Memorial Assoc., Westgate Street, Cardiff. |

### LIST OF SUBSCRIBERS.

| | |
|---|---|
| *DACRE, JOHN ... ... ... ... ... ... ... ... | 14 Eaton Crescent, Clifton. |
| *DACRE, R. I. ... ... ... ... ... ... ... ... | 14 Eaton Crescent, Clifton. |
| *DAVIES, E. D. D. ... ... ... ... ... ... ... ... | 6 Lansdown Place, Clifton. |
| *DAVIES, D. S., M.D. Lond., D.P.H. Cantab. ... | 6 Lansdown Place, Clifton. |
| *DAVIS, O. C. M., D.Sc., M.D. Brist. ... ... ... | 90 Cotham Brow, Bristol. |
| *DAWE, J. H., M.B. Edin. ... ... ... ... ... | 8 York Place, Clifton. |
| DENING, EDWIN ... ... ... ... ... ... ... ... | Manor House, Stow-on-the-Wold, Glos. |
| DEVINE, H., M.D Lond. ... ... ... ... ... | Corporation Mental Hospital, Portsmouth. |
| *DEVIS, H. F. ... ... ... ... ... ... ... ... | 160 Wells Road, Bristol. |
| *DIXON, T. B. ... ... ... .. ... ... ... ... | 134 Coronation Road, Bristol. |
| *DRAPES, T. L., M.B., B.C., B.A. Cantab. ... | St. Maur, Beaufort Square, Chepstow. |
| *DUNBAR, ELIZA L. WALKER, M.D. Zürich ... | 9 Oakfield Road, Clifton. |
| DYMOKE, F. ... ... ... ... ... ... ... ... | 21 Cotham Road, Bristol. |

| | |
|---|---|
| *EDGEWORTH, F. H., M.D., B.C., M.A. Cantab., D.Sc. Lond. ... ... ... ... ... ... ... ... | 13 Richmond Hill, Clifton. |
| EDWARDS, C. D., B.A., M.D. Cantab. ... ... | The Corderies, Chalford, Glos. |
| ELLIOT BROS. LTD. ... ... ... ... ... ... | *Australian Pharmaceutical Notes & News*, O'Connell Street, Sydney, N.S.W. |
| ELLIOTT, F. PERCY, M.B., C.M. Aberd. ... ... | 113 Grove Rd., Walthamstow, London, E.17. |
| *ELLIOTT, W. H. A., M.B., B.S. Lond. ... ... | Tudor Lodge, Cotham Brow, Bristol. |
| *EVERY-CLAYTON, L. E. V., M.D. Lond ... ... | The Old School House, Tetbury. |

| | |
|---|---|
| *FAILL, C. J. C. ... ... ... ... ... ... ... ... | 19 Portland Square, Bristol. |
| FARNFIELD, W. W. ... ... ... .. .. .. ... | Clive Vale, Gillingham, Dorset. |
| *FAWN, G. F. ... ... ... ... ... ... ... ... | 6 Oakfield Road, Clifton. |
| *FELLS, A., M.B., C.M. Edin. ... ... ... ... | 6 Elton Road, Clifton. |
| *FIRTH, J. L., M.D., M.S. Lond. ... ... ... .. | 8 Victoria Square, Clifton. |
| *FLEMMING, A. L., M.B., Ch.B. Brist. ... ... | 48 Pembroke Road, Clifton. |
| *FLEMMING C. E. S. ... ... ... ... ... ... | Manvers House, Bradford-on-Avon. |
| *FOSS, E. V., M.D., Ch.B. Brist. ... ... ... | Clouds Hill House, St. George, Bristol. |
| *FRASER, FORBES, *C.B.E.* ... ... ... ... ... | 5 The Circus, Bath. |
| *FULLER, R. A., *M.C.* .. ... ... ... ... ... | 2 Vane Street, Bath. |

| | |
|---|---|
| *GARDEN, R. R., M.A., M.B., B.Ch. ... ... ... | Guildhall, Bristol. |
| GARLAND, E. B., M.B., C.M. Edin. ... ... ... | 114*a* Hampton Road, Redland, Bristol. |
| GEE, C. A. H., M.B., Ch.B. Brist. ... ... ... | Evercreech, Somerset. |
| *GERRISH, D. S. ... ... ... ... ... ... ... | 328 & 330 Church Road, St. George, Bristol. |
| *GIBBS, A. N. GODBY ... ... ... ... ... ... | 52 Whiteladies Road, Clifton. |
| *GLENNY, E. T., M.B., B.S. Lond. ... ... ... | 102 Pembroke Road, Clifton. |
| *GORDON, R. G. M.D., B.Sc. Edin. ... ... ... | 6 Queen Square, Bath. |
| *GREEN, T. A., *D.S.O.*, M.D., C.M. Edin. ... | 33*a* Whiteladies Road, Clifton. |
| *GREER, W. J. ... ... .. ... ... ... .. ... | 19 Gold Tops, Newport Mon. |
| GRIFFITHS, CORNELIUS A. ... ... ... ... ... | 35 Newport Road, Cardiff. |
| *GRIFFITHS, J. S. ... ... .. ... ... ... ... | 20 Redland Park, Bristol. |
| *GROVES, E. W. HEY., M.D., M.S. Lond.... ... | 25 Victoria Square, Clifton. |

### LIST OF SUBSCRIBERS.

| | |
|---|---|
| *Hadfield, G., M.D. Lond. ... ... ... ... | General Hospital, Bristol. |
| *Hailes Clements D. G., M.D., C.M. Ed. ... | 2 Richmond Park Road, Clifton. |
| *Hall, E. George, M.B. Lond. ... ... ... ... | 42 Apsley Road, Clifton, Bristol. |
| *Hall, I. Walker, M.D., Ch.B.Vict. ... ... | Manilla Lodge, Clifton. |
| Handfield-Jones, Montagu ... ... ... ... | 50 Ashworth Mansions, W.C. 9. |
| *Harris, H. Elwin, B.A., M.B. Cantab. ... ... | 13 Lansdown Place, Clifton. |
| *Harsant, W. H. ... ... ... ... ... ... ... | Tower House, Clifton Down Road, Clifton. |
| *Harty, J. P. I., M.B., B.Ch., B.A.O., R.U.I. | West View, Clifton Down, Clifton. |
| *Hawkins, E. J. ... ... ... ... ... ... ... | Constantine House, Avonmouth. |
| *Herapath, C. E. K., *M.C.*, M.D. Lond. ... ... | Ormlie, Clifton. |
| *Herapath, C. K. C. ... ... ... ... ... ... | Stoneleigh, Cotham Park, Bristol. |
| *Hereford, C. F. A. ... ... ... ... ... ... | 12D Cotham Road, Bristol. |
| *Hill, Hedley, M.D. Durh. ... ... ... ... | 101 Pembroke Road, Clifton. |
| *Holland, W. A. L. ... ... ... ... ... ... | Pensions Hospital, Bath. |

Hospital, Bristol General (*Secretary*, T. W. Gregg).

| | |
|---|---|
| *Iles, A. E., *O.B.E.*, M.B., B.S. Lond. ... ... | 17 Victoria Square, Clifton. |

Infirmary, Bristol Royal (*Secretary*, E. C. Smith).

| | |
|---|---|
| Ingle, C. Durban ... ... ... ... ... ... ... | Somerton, Somerset. |

| | |
|---|---|
| *Jackman, W A. ... ... ... ... ... ... ... | 24 College Road, Clifton. |
| *Johnson, R. G., M.B., D.P.H. ... ... ... ... | 119 Redland Road, Bristol. |
| *Joll, C. A., M.B., M.S. Lond. ... ... ... ... | 21 Wimpole St., Cavendish Sq., W.1. |
| *Jones, J. Ellington ... ... ... ... .. ... | 82 Coldharbour Road, Bristol. |

| | |
|---|---|
| *Kennedy, W. P. M.D. Dubl. ... ... ... ... | 3 Gay Street, Bath. |
| *Kerfoot, Stanley J. ... ... ... ... ... ... | 109 East Street, Bedminster, Bristol. |
| *Kerr, R. A., *M.C.*, M.B., B.Ch. Belfast ... ... | 40 Orients Gardens, Belfast, Ireland. |
| *Kingston, S. H., M.B., Ch.B. Brist. . ... | 1 Richmond Hill, Clifton. |
| *Kyle, H. G., M.A., M.D. B.Ch. Oxon. ... ... | 31 Westbury Road, Bristol. |

| | |
|---|---|
| *Lace, F. ... ... ... ... ... ... ... ... ... | 5 Gay Street, Bath. |
| *Lansdown, R. G. P., M.D., B.S. Durh. ... ... | 39 Oakfield Road, Clifton. |
| *Lavington, C. C., M.B., B.S. Durh. ... ... | 1 Burlington Road, Redland, Bristol. |
| Lendon, A. A., M.D. Lond. ... ... ... ... | North Terrace, Adelaide, Australia. |
| *Lees, E. Leonard, M.D., C.M. Ed. ... ... ... | 1 Chesterfield Place, Clifton. |
| *Legat, R. Eddowes M.B., C.M. Edin.... ... | Murray House, Staple Hill, Bristol. |

### LIST OF SUBSCRIBERS.

| | |
|---|---|
| LEIGH, W. W. ... ... ... ... ... ... ... ... ... | Treharris, Glamorganshire. |
| *LEONARD, R. C., M.D. Durham ... ... ... ... ... | Bower Ashton, Bristol. |
| *LEVIS, J. S., *M.C.*, M.B., B.Ch., N.U.I. ... | 20 Gay Street, Bath. |
| *LEWIS, D. J. ... ... ... ... ... ... ... ... ... | 11 Gt. Bedford Street, Bath. |
| *LINTON, MARION S., M.B. Lond. ... ... ... | 21 Oakfield Road, Clifton. |
| *LISTER, L. MARGARET ... ... ... ... ... | 15 Park Place, Clifton. |
| *LOGAN, H. B. ... ... ... ... ... ... ... ... ... | Eastfield, Southville, Bristol. |
| *LUCAS, J. J. S., M.D. Lond. ... ... ... ... ... | 186 Wells Road, Totterdown, Bristol. |
| *LEGAT, R. E., M.B., C.M. ... ... ... ... ... | Murray House, Staple Hill. |

| | |
|---|---|
| MACKINNON, CHARLES, M.B., C.M., M.A. Glasg. | Davaar, Cirencester. |
| MACLEAN, EWEN J., M.D., C.M. Ed.... ... ... | 12 Park Place, Cardiff. |
| MACLEAN, J. CAMPBELL, M.B., C.M. Ed.... ... | Swindon House, Swindon. |
| *MACLEOD, DONALD, M.B., C.M. Ed. ... ... | 84 Redland Road, Redland, Bristol. |
| *MACWATTERS, J. C., *M.B.E.* ... ... ... ... | Almondsbury, near Bristol. |
| *MCKENZIE, W. G., *M.C.* ... ... ... ... ... | 101 Coronation Road, Bristol. |
| *MCLANNAHAN, J. G. ... ... ... ... ... .. ... | The Mount, Stonehouse. |
| MARSHALL, ARTHUR L., M.B., B.A. Cantab. ... | Thornleigh, Upper Clapton, London, E.5. |
| *MARTYN, G. J. KING, M.D., B.C., B.A. Cantab. ... ... ... ... ... ... ... ... ... | 8 Gay Street, Bath. |
| *MATHER, J. S., M.B., C.M. Aberd. ... ... ... | Lawrence Hill House, Bristol. |
| *MAYES, F. J. A. ... ... ... ... ... ... ... ... | 79 Pembroke Road, Clifton. |
| MIALL, C. L'O. ... ... ... ... ... ... ... ... | Elm Hayes, Paulton, near Bristol. |
| *MOORE, C. A., M.B., M.S. Lond.... ... ... ... | 56 St. Paul's Road, Clifton. |
| *MOORE, L. A., M.B., Ch.B. Brist. ... ... ... | 102 Redland Road, Bristol. |
| *MORRIS, A. G. ... ... ... ... ... ... ... ... | 18 Westbury Park, Westbury-on-Trym |
| MORRIS, L. N., M.B., Ch.B. Brist. ... ... ... | 24 All Hallows Road, Upper Easton, near Bristol. |
| *MORRIS, MARY E. H., M.D. Lond. ... ... ... | 19 Gay Street, Bath. |
| *MORTON, C. A. ... ... .. ... ... ... ... ... ... | 14 Vyvyan Terrace, Clifton. |
| *MUMFORD, W. G., M.B. Lond. ... ... ... ... | 18 The Circus, Bath. |
| *MYLES, G T. ... ... ... ... ... ... ... ... ... | 7 Apsley Road, Clifton. |

| | |
|---|---|
| *NEILD, NEWMAN, M.B., B.Ch. Vict. ... ... ... | 9 Richmond Hill, Clifton. |
| *NEWNHAM, W. H. C., M.B., M.A. Cantab. ... | 3 Lansdown Place, Victoria Square, Clifton. |
| *NICHOLS, F. C., *M.C.*, M.B., Ch.B. Brist. ... | 38 Whiteladies Road, Clifton. |
| *NIXON, J. A., *C.M.G.*, M.D. Cantab. ... ... | ... 7 Lansdown Place, Clifton. |
| *NORGATE, R. H. .. ... ... .. ... ... ... | Southmead Infirmary, Bristol. |

| | |
|---|---|
| *O'KEEFFE, D. S., M.B., B.Ch., R.U.I. ... ... | 12 Elton Road, Clifton. |
| OPPENHEIMER, SON & CO. LTD. ... ... ... | 179 Queen Victoria Street, London, E.C.4. |
| *ORMEROD, H. LAWRENCE, M.B., B.Ch. R.U.I. | 2 Henleaze Road, Westbury Park, Bristol. |
| *ORR-EWING, H. J., *M.C.*, M.D. Lond. ... ... | English Mission Hospital, Jerusalem. |

| | |
|---|---|
| *PARKER, GEORGE, M.D., M.A. Cantab. ... ... | 14 Pembroke Road, Clifton. |
| *PARKES, J. A., M.B., Ch.B. Liverpool. ... ... | 2 Effingham Road, Bristol. |
| PARKINSON, Major G. S., *D.S.O.*, R.A.M.C.... | M.O.H., Gibraltar. |

### LIST OF SUBSCRIBERS.

| | |
|---|---|
| *Parsons, H. F. ... ... ... ... ... ... ... | The Heath, Hazelwood Road, Sneyd Park, |
| Parsons, Sir J. H., M.B., B.S., D.Sc. Lond.... | 54 Queen Anne Street, London, W. |
| Paterson, Donald Rose, M.D., C.M. Ed. ... | 15 St. Andrew's Crescent, Cardiff. |
| *Pickering, C. F. ... ... ... ... ... ... ... | 13 Berkeley Square, Bristol. |
| *Pollard, J., M.B., B.Ch., B.A.O. ... ... ... | 52 Cumberland Road, Bristol. |
| Powell, J. J., M.D. Lond. ... ... ... ... ... | 2 Gloucester Road, Bishopston, Bristol. |
| Price, D. T. M.B. Lond. ... ... ... ... ... | Castle Cary, Somerset. |
| *Prichard, Arthur W. ... ... ... ... ... ... | 6 Rodney Place, Clifton. |
| *Pridie, H. H., M.B., C.M. Ed. ... ... ... | 17 Oakfield Road, Clifton. |
| *Prowse, Arthur B., M.D. Lond. ... ... ... | 5 Lansdown Place, Clifton. |

| | |
|---|---|
| *Rayner, D. C. ... ... ... ... ... ... ... ... | 9 Lansdown Place, Clifton, Bristol. |
| Ritchie, Thomas P. ... ... ... ... ... ... ... | 27 Oakfield Road, Clifton. |
| *Robertson, D., M.B. Ch.B. Edin. ... ... ... | 205 Wells Road, Knowle. |
| *Rogers, Beatrice, ... ... ... ... ... ... | Tower House, Whiteladies Road Clifton. |
| *Rogers, B. M. H., M.D., B.Ch., B.A. Oxon. ... | 14 Mortimer Road, Clifton. |
| *Rolfe, R., B.A., M.B., B.C. Cantab. ... ... | Park House, St. Andrews Road, Avonmouth. |
| *Rose, F. H. ... ... ... ... ... ... ... ... ... | 8 Chantry Road, Clifton. |
| *Rudge, C. King ... ... ... ... ... ... ... ... | 145 Whiteladies Road Bristol. |
| *Rutherford, J. M., M.B., C.M. Edin. ... ... | Brislington House, near Bristol. |

| | |
|---|---|
| *Salisbury, Walter, M.D., M.S. Lond. ... | 4 Durdham Park, Clifton. |
| *Shaw J. E., M.B., C.M., Edinb. ... ... ... | 23 Caledonia Place Clifton. |
| *Short, A. R., M.D., B.S., B.Sc. Lond. ... .. | Montrose House, Queen's Road, Clifton. |
| *Short, L. J., M.D., B.S. Lond., M.D. Brist. ... | Grove Park House, Weston-super-Mare. |
| *Smith, D. Munro ... ... ... ... ... ... ... ... | 73 Downs Park East, Bristol. |
| *Smith, G. Cockburn, M.D. Brux. ... ... ... | 12 South Road, Newton Abbot. |
| Smith, Rev. Reginald, M.A. Cantab. ... ... | The Vicarage, Walpole St. Andrew, Norfolk. |
| Smith, W. Steele, M.B. Lond. ... ... ... | Caldecote, Cambridge. |
| *Smith, W. Alexander, M.B., M.A. Oxon. ... | 70 Pembroke Road, Clifton. |
| *Smyth, Richard, M.A., M.D. Dub. ... ... | Burnley, Westbury-on-Trym. |
| Society, Cardiff Medical (*Hon. Lib.*, Queen Street, Cardiff). | |
| Society, Manchester Medical ... ... ... | Medical Society's Library, The Owens College, Manchester. |
| *Stack E. H. E., B.A., M.B., B.C. Cantab. ... | Arvalee, Clifton Down Road Clifton. |
| *Statham, R. S. S., *O.B.E.*, M.D., M.Ch. Brist. | 24 College Road, Clifton. |
| *Stock, W. S. V., M.B., B.S. Lond. ... ... ... | 1 Mortimer Road, Clifton. |
| *Swain, James, *C.B.*, *C.B.E.*, M.D., M.S. Lond. | 4 Victoria Square, Clifton. |
| *Swayne, W. Carless, M.D. Lond. ... ... ... | 2 Clifton Park, Clifton. |
| *Symes, J. O., M.D. Lond. ... ... ... ... ... | 71 Pembroke Road, Clifton. |
| *Smythe, H. J. D., *M.C.*, M.B., M.S. ... ... | 72 St. Paul's, Road, Clifton. |

### LIST OF SUBSCRIBERS.

| | |
|---|---|
| *Tasker, D. G. C., M.B., M.S. Lond. ... ... | Bristol General Hospital. |
| *Tate, Colonel G. W. *C.M.G.*, *D.S.O.*, M.B., B.Ch. Dub. ... ... ... ... ... ... ... | 9 Park Street, Bath. |
| Taylor, A. L. ... .. ... .. ... ... ... ... | The Royal Infirmary, Bristol. |
| *Taylor, H. N., M.A. Cantab., M.D. Dub. ... | Hillside, Ebbw Vale. |
| *Taylor, James ... ... ... ... ... ... ... ... | 36 Alma Road, Clifton. |
| *Temple, G. H., M.B., C.M. Ed. ... ... ... | Ailanthus, Weston-super-Mare. |
| Thin, James ... ... ... ... ... ... ... ... ... | 54 & 55 South Bridge, Edinburgh. |
| *Thomas, J. D., M.B. Cantab. ... ... ... ... | Northwoods, Winterbourne. |
| *Todd, A. T., *O.B.E.*, M.B., Ch.B. Edin. ... | Clayton, Clifton Park, Clifton. |
| *Trist, J. R. R., *M.C.* ... ... ... ... ... ... ... | 12 Alma Road, Clifton. |

| | |
|---|---|
| *Vickery, W. H. ... ... ... ... ... ... ... ... | 1 Trewartha Park, Weston-super-Mare. |

| | |
|---|---|
| *Walker, Cyril H., M.B., B.A. Cantab. ... ... | 8 Oakfield Road, Clifton. |
| *Wallace, John, *O.B.E.*, M.B., C.M. Edin. ... | Lauriston, Weston-super-Mare. |
| *Wallace, J. T., M.B., B.Ch., B.A. R.U.I. ... | 48 Coronation Road, Bristol. |
| *Walters, C. F. ... ... ... ... ... .. ... ... | 5 Mortimer Road, Clifton. |
| *Wanklyn-James, C. W., *O.B.E.* ... ... ... | 3 Mortimer Road, Clifton. |
| *Waterhouse, R., M.D. Lond. .. ... .. ... | 25 Circus, Bath. |
| *Watson-Williams, P., M.D. Lond.... ... ... | 2 Rodney Place, Clifton. |
| *Watson-Williams, E., *M.C.*, M.B., B.Ch., B.A. Cantab. ... ... ... ... ... ... ... | 12 Victoria Square, Clifton. |
| *Whicher, A. H. ... ... ... ... ... ... ... ... | 115 Queen's Road, Clifton. |
| Whitehouse, H. Beckwith, M.S. ... ... | 52 Newhall Street, Birmingham. |
| Wigan, C. A., M.D. Durh., J.P. ... ... ... ... | Deepdene, Portishead. |
| Wigmore, J., M.D. R. U. I. ... ... ... ... ... | 16 Queen Square, Bath. |
| *Wilkinson, S. H., M.D. Edin. ... ... ... | Pensions Hospital, Ashton Court. |
| *Willcox, J. W. J., M.B., B.S. Lond. ... ... | Glastonbury. |
| Willcox, R. Lewis ... ... ... ... ... ... ... | 97 London Road, Salisbury. |
| *Williams, E. C., M.B., B.C., B.A. Cantab. ... | 159 Whiteladies Road, Clifton. |
| *Williams, G. C., B.A. Cantab. ... ... ... | Ham Green, Pill. |
| Williams, H. Egerton ... ... ... ... ... | The Mount, Newport, Mon. |
| *Williams, L. H., M.D. Durh. ... ... ... ... | Thornbury, Glos. |
| Williams, S. R., M.B. Lond. ... ... ... ... | Buckfastleigh, Devon. |
| *Wills, W. K., M.B., B.C. Cantab. ... ... ... | 19 Whiteladies Road, Clifton. |
| *Windsor-Aubrey, H. W. ... ... ... ... ... | 79 Coronation Road, Bristol. |
| *Wood, Duncan ... ... ... ... ... ... ... | 14 Victoria Square, Clifton. |
| *Wood, W. V., *M.C.* ... ... ... ... ... ... | Henley Lodge, Yatton, Som. |
| *Woodcock, O. H., M.D., Ch.B. Man. ... ... | 46 College Road, Clifton. |
| *Wright, A. J., M.B., B.S. Lond. ... ... ... | 14 Victoria Square, Clifton. |

### Local Medical Notes.

UNIVERSITY OF BRISTOL.—Students of the University have recently passed the following examinations:—

CH.M. (*Gynæcology*).—R. S. S. Statham.

CH.M. (*Laryngology*).—E. Watson-Williams.

D.P.H. (*Part I. only*).—B. A. Astley-Weston.

M.B., Ch.B. Bristol.—W. K. A. Richards. *Final Examination* (*Part II. Completing Examination*): Phyllis Beames (2nd Cl. Hons.), M. Critchley (1st Cl. Hons.), Madge E. Golding, W. A. Jackman, Winifred G. Nott (2nd Cl. Hons.), P. Phillips (1st Cl. Hons.), Victoria S. Tryon. *Part I.* (*including Forensic Medicine and Toxicology*): B. A. Crook, Constance L. Griffiths, J. M. Evans, F. J. Hector, Marguerite G. Hughes, F. May Jones, A. J. Keevil, E. C. K. Kenderdine, Doris M. Pullen, J. A. L. Roberts, H. L. Shepherd. *Part I. only:* F. H. Bodman, Carrie H. Osmond, H. J. H. Spreadbury. *Second Examination* (*Part II.*): A. F. Alford, K. F. Alford, A. T. Binnie, G. W. R. Bishop, H. W. Brassington, D. E. Crellin, B. V. F. Dawkins, C. H. Durnford, F. S. Dymond, S. J. H. Griffiths, C. I. Ham, F. H. Hollingshead, J. A. Hooker, C. T. Hyatt, A. H. Lowther, R. E. Lucas, G. M. Minifie, W. S. Ormiston, B. Y. de la Pasture, M. P. Posthuma, D. C. Prowse, L. G. Robertson, E. S. Rogers, H. Rogers, H. J. Satchwell, J. F. Southward, S. P. Taylor, G. M. Terrell, M. M. Te Water, T. W. Ware, K. M. Willmore.

*Second Examination* (*Part I.*): A. H. Bright, A. P. Gorham.

*In Elementary Anatomy* (*Completing Examination*): J. F. O. Bodman, M. E. Drew.

*In Organic Chemistry only:* U. M. Hopkins.

Diploma in Dental Surgery.—*Final Examination:* W. Bayly, L. C. Bodey. *Third Examination* (*Dental Mechanics and Materia Medica*): J. F. Rigg. *Dental Mechanics* (*Completing Examination*): S. H. Bedford, W. B. Bawrey, M. P. Browning, H. P. Evans, G. J. Kelly, R. Lonnon, R. C. Price, J. R. Ritblatt, J. S. Roberts. *Dental Mechanics and Metallurgy only:* A. V. Harding. *In Dental Metallurgy only:* J. J. Gillard, R. G. Downes, D. Griffiths, B. Florence Taylor. *Second Examination:* F. J. Farr. *In Physiology and Histology and Anatomy* (*Completing Examination*): J. Evans, H. C. Hopes, K. A. Hunt, E. H. Longney, C. G. Payne, H. W. Pigeon, M. Ritblat, C. G. Saxon, C. R. Woods. *In Physiology and Histology and Anatomy only:* G. G. Royal. *In Dental Anatomy only:* V. E. N. Allen, H. N. V. Barnes, S. H. Bedford, A. S. C. Boulter, M. P. Browning, A. H. Butcher, R. G. Butterworth, L. H. Challenger, H. P. Evans, G. C. Friend, G. Holt, G. J. Kelly, R. C. Price, J. S. Roberts, W. H. Simpkiss, C. E. Smith, K. M. Watson, F. C. Winter.

B.D.S.—*Second Examination:* E. K. Tratman. *Final Examination:* G. N. Season.

L.D.S., R.C.S., Eng.—*Dental Mechanics:* C. E. Down, G. S. Williams. *Dental Metallurgy:* N. H. Simmonds. *Anatomy and Physiology:* D. J. Andrews, L. G. House.

F.R.C.S. Edin.—E. Watson-Williams.

### Bristol Medico-Chirurgical Society.

### President.

C. H. WALKER, M.B., B.A. Cantab.

### President-Elect.

J. A. NIXON, C.M.G., M.D. Cantab.

### Committee.

*Ex-Officio*
- L. E.V. EVERY-CLAYTON, M.D. Lond. (Retiring President).
- P. WATSON-WILLIAMS, M.D. Lond. (Editor of *Journal*).
- C. K. RUDGE (Hon. Librarian).
- A. L. FLEMMING, M.B., Ch.B. Brist. (Editorial Secretary).

*Elected.*

F. LACE } Ex-Presidents.
R. G. P. LANSDOWN, M.D., B.S. Durh. }
J. LACY FIRTH, M.D., M.S. Lond.
J. O. SYMES, M.D. Lond.
T. CARWARDINE, M.S. Lond.
D. C. RAYNER.
FORBES FRASER.
C. FERRIER WALTERS.

### Honorary Secretary.

S. V. STOCK, M.B., B.S. Lond.

Medical Practitioners wishing to join the Society are requested to communicate with the Hon. Secretary, Mr. S. V. STOCK, 1 Mortimer Road, Clifton. The Annual Subscription is £1 11s. 6d.

---

*Extracts from Journal By-laws.*

4.—The *Journal* shall be delivered free to Subscribers and also to Member of the Society whose subscriptions are not in arrear.

6.—The Annual Subscription to the *Journal* is 10/6.

---

Communications intended for insertion in the *Journal* should be sent to the Editor, Dr. P. WATSON-WILLIAMS, 2 Rodney Place, Clifton, Bristol.

Books for Review and Exchange Journals should be sent to the Assistant-Editor, *Bristol Medico-Chirurgical Journal*, Medical Library, The University, Bristol.

Communications referring to the delivery of the *Journal* should be sent to the Editorial Secretary, A. L. Flemming, 48 Pembroke Road, Clifton, Bristol, to whom Subscriptions are to be paid *in advance*.

Cases for Binding Vols. I—XXXVIII are now ready, and may be obtained, price 1/9 each (postage extra), upon application to J. W. ARROWSMITH LTD., Quay Street, Bristol; or the *Journal* will be bound including Case, for 7/6 per volume.

### *PUBLICATIONS RECEIVED.*

From Baillière, Tindall and Cox, London :
*Practical Psycho-analysis.* By H. Somerville, B.Sc., M.R.C.S., L.R.C.P. 1922. Price 6s.
*Aachen Treatment of Syphilis.* By Reginald Hayes, M.R.C.S. Fourth Edition, revised. 1922.

From John Bale, Sons and Danielsson, Ltd., London :
*Transactions and Tenth Annual Report of the London Dermatological Society.* 1921. Price 2s. 6d.
*The Venereal Clinic.* Edited by Ernest R. T. Clarkson, M.A., M.R.C.S. 1922. Price 25s. net.
*A Compendium of the Pharmacopœias.* By C. J. S. Thompson, M.B.E. Sixth Edition. 1922. Price 10s. net.
*A Study of Intestinal Stasis.* By J. C. Watt, M.C., M.B., Ch.B. 1922. Price 1s. net.

From Cassell and Company, Ltd., London :
*Tumours: Innocent and Malignant.* By Sir John Bland-Sutton, LL.D., F.R.C.S. Seventh Edition. 1922. Price 30s. net.

From W. J. Dornan, Philadelphia :
*Transactions of the College of Physicians of Philadelphia.* Volume XLII. 1920.
*Medical and Surgical Reports of the Episcopal Hospital.* Volume V. 1920.

From Dr. M. Ferré, Pau :
*Fonctionnement d'une Maternité Départementale (Thése).* Par Marcel Ferré. 1921.

From Henry Frowde and Hodder and Stoughton, London :
*A Manual of Fevers.* By Claude Buchanan Ker, M.D. [Second Edition.] [1922.] Price 12s. 6d.
*Hyperpiesia and Hyperpiesis.* By H. Batty Shaw, M.D. [1922.] Price 21s.
*Guy's Hospital Reports.* Vol. LXXI. No. 1, January, 1922. Price 12s. 6d. net.
*The Principles of Electrotherapy.* By W. J. Turrell, M.A., D.M., B.Ch. [1922.] Price 12s. 6d. net.
*Treatment of Injuries of the Peripheral Spinal Nerves.* By Sir Harold J. Stiles, K.B.E., F.R.C.S. Ed., and M. F. Forrester-Brown, M.S., M.D. 1922. Price 15s. net.
*Blood Transfusion.* By Geoffrey Keynes, M.A., M.D. [1922.] Price 8s. 6d. net.
*Reports of the St. Andrew's Institute for Clinical Research, Fife.* Volume I. [1922.] Price 10s. 6d. net.

From Government Printing Office, Washington :
*Annual Report of the Smithsonian Institution for* 1919. 1921.

From William Heinemann (Medical Books) Ltd., London :
*Manual of Physio-Therapeutics.* By Thomas Davey Luke, M.D. New and Revised Edition. 1922. Price 25s. net.

From H. K. Lewis and Co., Ltd., London :
*The Relations of Tuberculosis to General Bodily Conditions.* By F. Parkes Weber, M.A., M.D., F.R.C.P. 1921. Price 2s. 6d. net.

From E. and S. Livingstone, Edinburgh :
*The Mechanism of the Brain.* By Professor Leonardo Bianchi. Authorised Translation by James H. Macdonald, M.B. With a Foreword by Professor C. Lloyd Morgan. 1922. Price 21s. net.

From E. Merck, Darmstadt :
Merck's *Jahrberichte über neuerungen auf den gebieten der Pharmako-Therapie und Pharmazie.* XXXIII. und XXXIV. Jahr. 1921.

From John Murray, London :
*Saint Bartholomew's Hospital Reports,* 1919. Volume LIV. 1922.

From The Psychiatric Institute, Morristown, N.J. :
*The Journal of Metabolic Research.* Volume I., No. 1. January, 1922.

From University of London Press, London :
*Intrinsic Cancer of the Larynx and the Operation of Laryngo-fissure.* By Irwin Moore, M.B., C.M. 1921. Price 20s. net.

From John Wright and Sons Ltd., Bristol :
*An Index of Treatment.* By Various Writers. Edited by Robert Hutchison, M.D., F.R.C.P., and James Sherren, C.B.E., F.R.C.S. Eighth Edition, revised and enlarged. 1921. Price £2 2s. 0d. net.
*Functional Nervous Disorders.* By Donald E. Core, M.D. 1922. Price 25s. net.
*A Synopsis of Medicine.* By Henry Letheby Tidy, M.A., M.D. Second Edition, revised. 1922. Price 21s. net.
*The Medical Annual,* 1922. Price £1 net.

